# Draft Genome Sequences of *Rhodosporidium toruloides* Strains ATCC 10788 and ATCC 10657 with Compatible Mating Types

**DOI:** 10.1128/genomeA.00098-16

**Published:** 2016-03-10

**Authors:** Jie Hu, Lianghui Ji

**Affiliations:** Temasek Life Sciences Laboratory, National University of Singapore, Singapore

## Abstract

*Rhodosporidium toruloides* ATCC 10788 (haploid, A1 mating type) and ATCC 10657 (haploid, A2 mating type) were derived from the same diploid parent strain *Rhodotorula glutinis* ATCC 90781 and are important strains for metabolic engineering. Draft genome sequences of both strains are reported here. The current assembly of strain ATCC 10788 comprises 61 scaffolds with a total size of 20.75 Mbp and a GC content of 62.01%, while that of strain ATCC 10657 comprises 137 scaffolds with a total size of 21.49 Mbp and a GC content of 61.81%. Genome annotation predicts 7,730 and 7,800 protein encoding genes for strain ATCC 10788 and strain ATCC 10657, respectively.

## GENOME ANNOUNCEMENT

*Rhodosporidium toruloides* has attracted increasing interest since the 1980s because of its capability for high-cell-density fermentation and high-level lipid production (1–3). It is a promising production host for renewable fuels and chemicals. Four genome sequences of *R. toruloides* strains have been published since 2012, i.e., MTCC 457 (4), NP 11 (5), CECT 1137 (6), and ATCC 204091 (previously *Rhodotorula glutinis*) (7). Haploid strains ATCC 10788 and ATCC 10657, obtained from the American Type Culture Collection, were derived from the same parent strain *Rhodotorula glutinis* ATCC 90781 with A1 and A2 mating types, respectively, and have been targets for metabolic engineering for the production of high-value bioproducts (8–10).

Whole-genome sequencing was carried out by Macrogen, Inc. (Republic of Korea) with the Illumina HiSeq 2000 platform using paired-end (insert length of 200 bp) and mate-pair (10-kb insert) libraries. Approximately 5 Gb of raw data (101-bp reads with about 100× sequencing depth) were generated from each strain. Several *de novo* assemblies, like SOAPdenovo (11), ALLPATHS-LG (12), CLC genomics workbench (Qiagen), Velvet (13), ABySS (14), IDBA-UD (15), and MaSuRCA (16), were used to perform the assembly. The best assemblies (by ALLPATHS-LG) were evaluated and chosen by the quality assessment tool for genome assemblies (QUAST) (17). The genes were predicted by GeneMark-ES (18) and MAKER2 (19). Gene functions and evolutionary relationship were identified by BLAST (20) against the NCBI nonredundant databases (nt and nr).

The draft genome sequence of ATCC 10788 comprises 61 scaffolds with a total size of 20.75 Mbp and a GC content of 62.01%, while that of strain ATCC 10657 comprises 137 scaffolds, with a total size of 21.49 Mbp and a GC content of 61.81%. A total of 7,730 genes for strain ATCC 10788 and 7,800 genes for strain ATCC 10657 were predicted by GeneMark-ES without a reference annotated genome, whereas 7,181 and 7,085 for ATCC 10788 and ATCC 10657, respectively, were predicted by MAKER2 based on the *Rhodotorula glutinis* ATCC 204091 protein database. A comparison of the genome assemblies to published ones by QUAST (genome fraction %) reveals that 98.78 to 99.63% of contig bases of ATCC 10788 could be aligned to the genome of MTCC 457 (4), NP 11 (5), or CECT 1137 (6), while 0.14% could be aligned to the genome of ATCC 204091 (7). On the other hand, 99.51% of the contig bases of ATCC 10657 could be aligned to the genome of ATCC 204091, but 0.11 to 0.16% could be aligned to the genome of MTCC 457 (4), NP 11 (5), or CECT 1137 (6). These data suggest that *R. toruloides* of different mating types have diversified extensively in nucleotide sequences and gene organizations.

### Nucleotide sequence accession numbers.

This whole-genome shotgun project has been deposited at DDBJ/EMBL/GenBank under the accession numbers LNQQ00000000 and LNKU00000000.
